# Hepatitis Vaccines: Recent Advances and Challenges

**DOI:** 10.3390/vaccines13111174

**Published:** 2025-11-20

**Authors:** Mei Lu, Yakun Liu, Lele Li, Xueke Liu, Bin Wu, Yingping Wu

**Affiliations:** 1 Department of Clinical Laboratory, the Fourth Affiliated Hospital of School of Medicine, and International School of Medicine, International Institutes of Medicine, Zhejiang University, Yiwu 322000, China; lumei@zju.edu.cn (M.L.); lilele9411@zju.edu.cn (L.L.); 8020050@zju.edu.cn (X.L.); 2 Department of Cardiothoracic Surgery, the Fourth Affiliated Hospital of School of Medicine, and International School of Medicine, International Institutes of Medicine, Zhejiang University, Yiwu 322000, China; 8020041@zju.edu.cn; 3 Jinhua Center for Disease Control and Prevention, Jinhua 321000, China

**Keywords:** hepatitis vaccines, vaccine design, innovative adjuvants, immune responses, immune evasion

## Abstract

Viral hepatitis constitutes a substantial global public health challenge. The etiological agents, referred to as hepatitis viruses, are primarily categorized into five types: hepatitis A virus (HAV), hepatitis B virus (HBV), hepatitis C virus (HCV), hepatitis D virus (HDV), and hepatitis E virus (HEV). Among the various preventive strategies, vaccination is widely acknowledged as the most cost-effective and efficient method for controlling viral hepatitis and its related hepatic complications. To date, numerous countries have initiated extensive vaccination programs targeting hepatitis A and hepatitis B. Advances in biotechnology have facilitated substantial progress in vaccine formulation design, the development of innovative adjuvants, and the utilization of novel vectors. However, significant challenges persist, including inadequate vaccination coverage, inconsistent immune responses among vulnerable populations, and concerns regarding vaccine safety. This article presents a systematic review of recent advancements, the current status of vaccination efforts, and ongoing challenges associated with hepatitis vaccines, with the objective of providing critical insights to support the World Health Organization’s goal of eliminating viral hepatitis as a public health threat by 2030.

## 1. Introduction

WHO statistics show that deaths from viral hepatitis rose from 1.1 million in 2019 to 1.3 million in 2022 across 187 countries, significantly threatening global public health [1]. Viral hepatitis is caused by five primary hepatotropic viruses: HAV, HBV, HCV, HDV, and HEV. HAV and HEV are primarily transmitted via the fecal-oral route and typically cause a self-limited, acute hepatitis that generally require only symptomatic and supportive management. In contrast, HBV and HCV infections are major contributors to chronic hepatitis, liver cirrhosis, and hepatocellular carcinoma (HCC). HDV, a defective virus, requires co-infection or superinfection with HBV and is associated with an increased risk of severe liver disease, including cirrhosis and hepatocellular carcinoma. Currently, safe and effective vaccines are available for the prevention of hepatitis A, B, and E; antiviral therapies exist for HCV and HDV infections, although no vaccine is yet available for these two types.

Vaccines constitute one of the most efficacious medical interventions for combating viral hepatitis. They are primarily classified into preventive and therapeutic categories, both designed to elicit specific immune responses through the administration of non-infectious antigens, thereby either preventing infection or modulating the progression of the disease. Similar to natural infections, vaccines work by initiating the innate immune response, which subsequently triggers the antigen-specific adaptive immune response. Innate immunity is rapidly activated upon pathogen exposure but lacks antigenic specificity. In contrast, adaptive immunity develops during the later stages of infection and is characterized by a diverse population of lymphocytes and antibodies capable of recognizing and eliminating virtually all known pathogens. Vaccines contain specific antigens derived from pathogens. Following vaccination, these antigens induce cell-mediated immunity through the activation of highly specific T lymphocyte subsets and stimulate humoral immunity by promoting B lymphocyte production of pathogen-specific antibodies [2]. With continuous advancements in biotechnology, a variety of innovative vaccine platforms have been developed to improve immunogenicity and safety. These platforms include protein/peptide-based vaccines, DNA vaccines, and mRNA vaccines, alongside the implementation of novel delivery systems and adjuvants [3,4]. Considering that hepatitis A and E infections generally resolve spontaneously and that effective preventive vaccines are already available, contemporary research initiatives have increasingly concentrated on the development of vaccines for hepatitis B and C. Although the hepatitis B preventive vaccine has been successfully deployed worldwide and has shown high efficacy, there is now a heightened focus on creating vaccines with higher immunogenicity for low responders, in addition to therapeutic vaccines for individuals who are already chronically infected with HBV.

This review provides an overview of currently approved viral hepatitis vaccines and those undergoing clinical development. Despite notable advancements in the development of these vaccines in recent years, several challenges persist. Through an extensive analysis of existing literature, we examine critical issues such as vaccination coverage, as well as immunogenicity, efficacy, and safety in specific populations. Additionally, we propose potential strategies to improve vaccine efficacy and delineate future research directions, with the objective of contributing a scientific basis for achieving the WHO’s goal of eradicating viral hepatitis as a public health threat by 2030.

## 2. Hepatitis A Vaccine

### 2.1. Hepatitis A

In contrast to hepatitis B and C, hepatitis A is an acute infectious disease caused by HAV. It typically does not progress to chronic liver disease; however, it can result in a range of clinical manifestations, from mild symptoms to severe illness. It is estimated that over 100 million HAV infections occur globally each year, leading to approximately 15,000 to 30,000 deaths annually [5]. The epidemiological patterns of hepatitis A exhibit considerable regional variation, with a notably higher prevalence in low- and middle-income countries characterized by inadequate sanitary conditions and suboptimal hygienic practices. In these regions, up to 90% of children contract the HAV by the age of 10. This high prevalence is closely associated with insufficient sanitation infrastructure and limited public health interventions [6].

The predominant mode of HAV transmission is via the fecal-oral route, primarily through the consumption of contaminated food or water. In developing nations, inadequate hygiene practices elevate the probability of food and water contamination, thereby exacerbating the risk of HAV dissemination [6]. Therefore, enhancing the quality of drinking water and instituting comprehensive food safety protocols are essential for mitigating HAV transmission. Additionally, HAV can be transmitted through sexual contact, particularly among men who have sex with men (MSM), underscoring the necessity for targeted prevention strategies within high-risk populations [7]. In low endemicity regions, HAV infections predominantly occur among specific high-risk populations, including MSM, injecting drugs users, and travelers to regions of high endemicity who have not been previously vaccinated. Conversely, in countries with intermediate endemicity, there is an increased susceptibility and incidence of the disease among older age cohorts. In areas with high endemicity, up to 90% of children are infected before the age of 10 years [8].

At present, no specific antiviral therapy exists for hepatitis A; however, the disease can be effectively prevented through vaccination. The hepatitis A vaccine elicits protective antibody responses and establishes long-term immunological memory, facilitating a rapid immune response upon subsequent exposure. This significantly reduces both transmission rates and the overall disease burden. Consequently, advancing the development and widespread implementation of hepatitis A vaccination is a critical objective in global public health efforts.

### 2.2. Types of Hepatitis A Vaccine

Two main types of hepatitis A vaccines are currently in use: inactivated hepatitis A vaccine (HepA-I) and live attenuated hepatitis A vaccine (HepA-L) (Table 1). Hep-I is widely used globally, while HepA-L is mainly applied in China, and is also available in India, Guatemala, the Philippines, and Thailand [9].

#### 2.2.1. HepA-I

Inactivated vaccines are vaccines made from inactivated pathogens, typically achieved by using chemical or physical methods to destroy the virus’s ability to replicate but still retain their immunogenicity in order to trigger an immune response against specific pathogens, thus protecting the body from infection [10]. HepA-I is widely used globally, with its development history dating back to the 1990s.The first licensed HepA-I was Havrix, developed by GSK in 1992. It was made by culturing the HAV HM-175 strain in a cell culture medium, followed by inactivation with formalin. Aluminum hydroxide was subsequently added as an adjuvant to enhance immunogenicity [5]. It functions by adsorbing viral antigens and prolonging the interaction between antigens and immune cells, thereby enabling sustained stimulation of the immune system and enhancing the immune response [11]. Other globally used HepA-I include Vaqta, Avaxim, and Epaxal [12]. Furthermore, Chinese-manufactured vaccines such as Healive (TZ284), Weisairuian (LV8), and Veraxim (YN5) are also available. Notably, Veraxim is the first HepA-I developed using Vero cell culture [9]. Moreover, Aimmugen has been licensed for use in Japan but is not available in other countries or regions [13].

The widely used HepA-I is administered intramuscularly according to a schedule that consists of two doses. The primary dose is typically administered between 12 and 18 months of age, with the second dose recommended 6 to 18 months after the initial vaccination [14]. In contrast, the standard regimen for Aimmugen consists of three doses administered at 0, 2–4 weeks, and 6 months [13]. The promotion of hepatitis A vaccination has significantly reduced the incidence of hepatitis A. Since the introduction of HepA-I in 1995, the incidence of hepatitis A in the general population of the United States has declined by 95% [15]. A 20-year follow-up study demonstrated that over 97% of participants who received two doses of Havrix maintained seropositive anti-HAV antibody levels at the end of the follow-up period, regardless of whether the doses were administered on a 0–6 or 0–12 month schedule [16]. Another 20-year follow-up study showed that two doses of Epaxal provided protective immunity against HAV infection for at least 30 years in more than 95% of healthy recipients (mean age: 44.71 years) [17]. Collectively, extensive evidence supports the long-term protective efficacy of HepA-I against hepatitis A.

#### 2.2.2. HepA-L

Live attenuated vaccines employ weakened strains of live viruses that are capable of inducing a robust immune response without causing clinical disease. A major advantage of this vaccine type is its ability to closely mimic natural infection, thereby activating multiple arms of the immune system and generating a broad and durable immune response. Additionally, these vaccines typically require only a single or low dose to achieve long-term immunity [18]. The HepA-L produced in China, including Weisairuiji produced using the H2 strain and Havac produced using the LA-1 strain, is mainly used in China and India. It was initially formulated as liquid forms and later transitioned to freeze-dried forms in 2000 to improve its heat stability and facilitate storage and distribution [9].

HepA-L is administered using a subcutaneous single-dose immunization schedule for children aged 18 months and older. A single vaccine dose of 0.5 mL is recommended for children, and 1.0 mL for adults [14]. Extensive evidence indicates that HepA-L can induce long-term immunogenicity and sustained protective efficacy in vaccinated individuals [19]. A particularly representative example is a 10-year follow-up study of children aged 1 to 15 years (*n* = 30,908) who received a single dose of HepA-L (H2 strain), which showed that the anti-HAV IgG seropositivity rate at the end of the follow-up period was 80.2% [20]. In this cohort, a subgroup of 220 individuals initially aged 1–3 years underwent an extended follow-up period of up to 15 years, maintaining a final anti-HAV IgG seropositivity rate of 81.3% [21].This vaccine offer several advantages, including low cost, single-dose administration, high protective efficacy, and prolonged immunity that may be further enhanced through asymptomatic natural boosting. No major safety concerns associated with HepA-L during clinical trials or post-marketing surveillance. However, consistent with other live attenuated vaccines, HepA-L is not recommended in pregnant women or immunocompromised individuals.

### 2.3. Challenges of Hepatitis A Vaccine Administration

With improvements in hygiene conditions and practices, as well as the implementation of hepatitis A vaccination programs-particularly universal mass vaccination (UMV)-the transmission of HAV has significantly declined. However, the high cost of vaccines has impeded larger-scale implementation of a two-dose HAV vaccine strategy in most endemic countries in need for many years. The use of HepA-L can significantly reduce vaccination costs. However, regulatory approval for HepA-L remains limited to only a few countries, thereby hindering the global implementation of UMV programs. Evidence indicates that a single dose of the inactivated hepatitis A vaccine (Epaxal), administered to 105 children with a median age of 3.6 years, resulted in a serum anti-HAV concentration of 80 mIU/mL after 7.5 years [22], a level considered protective. This demonstrates that a single dose of HepA-I can elicit a durable immune response sufficient for protection. Therefore, in regions with insufficient vaccine coverage, adopting a single-dose UMV strategy using inactivated vaccines may represent a cost-effective approach to expand immunization coverage or at least recommend that high-risk groups receive single-dose vaccinations.

Apart from economic factors, public awareness of vaccination also significantly influences the vaccination rate. Although hepatitis A vaccination is recommended for unvaccinated travelers to high or intermediate HAV endemicity, compliance with this recommendation is not universal. This has led to travelers, especially susceptible ones, contracting hepatitis A even when traveling to low endemicity. Therefore, providing hepatitis A vaccination before departure is crucial for reducing hepatitis A related to travel, regardless of the destination, and should be offered as part of pre-travel consultations for all travellers [23]. A large European MSM Internet Survey (EMIS-2017) showed that among 113,884 MSM respondents, only 45% had received the hepatitis A vaccine, including 5% who received only one dose [24]. In recent years, hepatitis A outbreaks among MSM have been reported in Europe, Asia, Latin America, and North America. Effective control of these outbreaks requires comprehensive public health education interventions, along with enhanced vaccination coverage within the MSM population [25].

In addition, immunocompromised individuals, including people living with HIV(PLWH) and those receiving immunosuppressive drugs, are more susceptible to HAV infection, which tends to result in more severe clinical outcomes. Therefore, this population warrants special attention. A prospective single-center cohort study showed that among PLWH, patients undergoing immunosuppressive monotherapy or combination therapy, and healthy individuals, the seroconversion rates (SCR) two months after a single dose of HepA-I was 58%, 53%, 35%, and 94%, respectively. Compared with the control group, immunocompromised individuals exhibited substantially lower SCR. SCR measured at 6 months post-vaccination reached 97%, 88%, 69%, and 97%, respectively. Notably, only patients on combination immunosuppressive therapy showed a significantly lower SCR compared to healthy controls. Furthermore, 13 individuals experienced seronegative (<20 mIU/mL) during follow-up, including 2 from the PLWH group, 1 from the monotherapy group, and 10 from the combination therapy group. These 13 individuals received booster doses, of whom six achieved seroconversion. All non-responders following booster vaccination belonged to the combination therapy group, three of whom remained unresponsive throughout the entire study period. Based on these findings, it is evident that a single dose of HepA is insufficient to ensure protective immunity in immunocompromised individuals. It is recommended that these individuals receive a complete vaccine schedule or at least conduct antibody assessment. Post-vaccination serological assessment may only be necessary for patients receiving combination immunosuppressive therapy to determine if booster vaccination is needed [26].

Overall, it is essential to enhance public awareness regarding hepatitis A vaccination. Where resources allow, efforts should be made to maximize vaccination coverage. Particular emphasis should be placed on immunocompromised individuals, with a focus on strengthening antibody testing within this group. A thorough evaluation of long-term immune protection following vaccination, along with the potential need for booster doses, is necessary to prevent increases in HAV-related hospitalizations and mortality. To reduce the incidence of hepatitis A and ultimately achieve its elimination, strategies must extend beyond merely increasing vaccine coverage. Enhancing hygiene practices, ensuring access to clean drinking water, and guaranteeing food safety are essential measures for mitigating the transmission of foodborne infectious diseases, including hepatitis A.

## 3. Hepatitis B Vaccine

### 3.1. Hepatitis B

In 2022, an estimated 254 million individuals were living with chronic hepatitis B worldwide [27]. HBV infection can lead to severe hepatic complications, including cirrhosis, liver failure, and hepatocellular carcinoma. According to WHO, approximately 1.1 million people died from hepatitis B-related causes in 2022 [27]. In response to this burden, WHO has implemented a global strategy for viral hepatitis, aiming to eliminate viral hepatitis, particularly hepatitis B, as a public health threat by 2030.

HBV is transmitted through exposure to infected blood and body secretions. The primary routes of transmission include mother-to-child and male-to-male sexual contact [28]. Mother-to-child transmission can occur prenatally during pregnancy, intrapartum during delivery, or postnatally through close household contact, such as breastfeeding. In high-prevalence regions, perinatal transmission at birth is responsible for more than 50% of HBV infections, with 1–9% of infants born to HBV-positive mothers acquiring HBV in early life [29]. Mothers seropositive for hepatitis B e antigen (HBeAg) or who have high HBV DNA levels in their blood (>200,000 IU/mL) are at high risk of transmitting the infection to their infants in the absence of prophylaxis. Furthermore, infants infected with HBV within the first six months of life have a 90% probability of developing chronic infection. Although intrauterine transmission can occur, it is rare and typically associated with antepartum hemorrhage or placental tears [30]. In adolescence and adulthood, the primary modes of HBV transmission include unsafe sexual practices, particularly among unvaccinated MSM and heterosexual individuals with multiple sexual partners or those who engage with sex workers [30]. Additionally, injecting drug and other blood-related exposures, such as tattooing, acupuncture, and the inadequate sterilization of medical instruments, also contribute significantly to HBV transmission [30,31].

Current first-line therapeutic options for chronic hepatitis B (CHB) include pegylated interferon (PEG-IFN) and oral nucleoside analogues (NAs), such as entecavir (ETV), tenofovir disoproxil fumarate (TDF), and tenofovir alafenamide (TAF) [32]. In contrast to PEG-IFN, NAs are more commonly prescribed due to their favorable safety profile and convenient oral administration [28]. Although these antiviral therapies effectively suppress HBV replication in most patients, long-term treatment adherence remains suboptimal, with an overall rate of 74.6% [32]. Moreover, functional cure, defined as sustained hepatitis B surface antigen (HBsAg) loss or seroconversion based on assays with a lower limit of HBsAg detection of 0.05 IU/mL, is rarely attained [33]. A large international multicenter cohort study of patients with newly diagnosed CHB reported that only 2.1% achieved HBsAg loss after 10 years of ETV or TDF therapy [34]. Consequently, the development and widespread implementation of preventive and therapeutic vaccines have become critical priorities in the effort to improve clinical outcomes.

### 3.2. Preventive Hepatitis B Vaccines

#### 3.2.1. Plasma-Derived Hepatitis B Vaccine

The first-generation hepatitis B vaccine is a plasma-derived vaccine produced by inactivating and purifying hepatitis B surface antigen (HBsAg) extracted from the blood of HBV carriers. Widely available examples include Heptavax B manufactured by Merck, and Hevac B produced by Institut Pasteur [35]. This generation of vaccine demonstrates strong immunogenicity and effectively induces anti-HBs antibodies, thereby conferring protective immunity. However, it is associated with high costs, a prolonged manufacturing process, and a potential risk of blood-borne pathogen transmission due to its human plasma origin. Consequently, it has been superseded by recombinant hepatitis B vaccine.

#### 3.2.2. Recombinant Hepatitis B Vaccine

With the advent of DNA recombination technology, scientists have developed safer and more efficient methods for vaccine production. Using genetic engineering techniques, the gene fragment encoding HBsAg, known as the S gene, is isolated and inserted into the genome of yeast cells. The yeast cells are then grown through a fermentation process to produce HBsAg protein. Subsequently, the HBsAg polypeptides obtained after extraction and purification are adsorbed onto aluminum hydroxide, which acts as an adjuvant to enhance the immunogenicity of the vaccine [3]. Merck’s Recombivax HB, a recombinant hepatitis B vaccine based on yeast, was the first to be licensed for clinical use in 1986. In December of the same year, GSK’s Engerix-B also obtained approval. Compared with plasma-derived vaccines, recombinant vaccines offer significantly improved safety profiles and more effective control of costs. As a result, they have become the predominant type of hepatitis B vaccine used globally.

The recombinant hepatitis B vaccine is typically administered according to a three-dose schedule at 0, 1, and 6 months, respectively. The widespread administration of the timely birth dose (TBD) has proven highly effective in preventing mother-to-child transmission of HBV. Since the implementation of the universal hepatitis B vaccination program in Taiwan, China, in 1984 the prevalence of HBsAg among newborns has significantly declined from 7.7% to 0.4% [36]. The standard three-dose schedule provides long-term protection, with evidence indicating sustained efficacy for over 30 years post-vaccination [37]. For high-risk populations, particularly infants born to mothers who are positive for HBV, the administration of hepatitis B immunoglobulin (HBIG) in conjunction with vaccination is recommended to augment immune protection. Studies have demonstrated that co-administration of HBIG and the hepatitis B vaccine within 12 to 24 h after birth markedly reduces mother-to-child transmission. Among infants born to HBeAg-negative mothers, the transmission rate has decreased from 10–30% to nearly 0%; among those born to HBeAg-positive mothers, it has been reduced from 70–90% to 4–12% [38].

The hepatitis B vaccine is regarded as one of the most successful viral vaccines globally. Since its introduction in the early 1980s, it has been implemented extensively across numerous countries and regions. By the end of 2024, 190 countries had incorporated the hepatitis B vaccine into their national infant immunization programs, with a global coverage rate of approximately 84% for the full three-dose vaccination series [39]. The overall response rate to the HBV vaccine, defined as the proportion of individuals achieving anti-HBs antibody levels exceeding 10 mIU/mL, ranges from 90% to 95% among vaccinated individuals [40]. In China, the current vaccination coverage has reached 99.6%, with a timely birth dose administration rate of 95.6%. Through complete immunization of newborns combined with antiviral therapy for pregnant women with HBV infection, the mother-to-child transmission rate in China has decreased to 0.23% [41]. These data strongly indicate that vaccination effectively prevents HBV infection. Furthermore, a 37-year follow-up study demonstrated that the HBV vaccine provides 72% protection against the development of hepatocellular carcinoma and 70% effectiveness in reducing liver cancer-related mortality among vaccinated infants [42]. This indicates that vaccination with the HBV vaccine also significantly reduces long-term mortality associated with chronic liver diseases.

#### 3.2.3. Next-Generation Recombinant Hepatitis B Vaccine

In addition to the S protein, the hepatitis B virus surface contains two other key antigens: pre-S1 and pre-S2 proteins. Recombinant vaccines including pre-S proteins are primarily designed as multi-epitope vaccines that comprise the pre-S1, pre-S2, and S antigens. These vaccines exhibit enhanced immunogenicity and induce antibody more rapidly compared to traditional vaccines. PreHevbrio (also known as Sci-B-Vac or PreHevbri) is the first licensed three-antigen hepatitis B vaccine (TAV), produced in Chinese hamster ovary (CHO) cells capable of expressing all three HBV envelope proteins and formulated with aluminum hydroxide as an adjuvant. It received approval in 2021 in multiple regions, including the United States, the European Union, the United Kingdom, and Canada, for the prevention of HBV infection in adults aged 18 years and older. The standard vaccination schedule consists of three doses, with the first dose administered intramuscularly at any time, followed by the second and third doses at one month and six months after the first dose, respectively [43]. Clinical studies have demonstrated that after receiving the 0-1-6 three-dose vaccination schedule of PreHevbrio, the proportion of high responders (defined as anti-HBs ≥ 100 mIU/mL) was 81.4% two months after the second dose and increased to 97.6% one month after the third dose, and the geometric mean concentrations (GMCs) of HBs antibodies were 413.6 mIU/mL and 6799.9 mIU/mL, respectively. These results indicate that PreHevbrio elicits robust seroprotection. Furthermore, a seroprotection (anti-HBs ≥ 10 mIU/mL) rate of 98.8% was observed two months after the second dose, suggesting that two doses of PreHevbrio are sufficient to confer high-titer antibody responses and achieve seroprotection in over 95% of recipients [44]. The immunogenicity of PreHevbrio is consistent across all adult age groups, including individuals over 45 years of age and other populations known to exhibit diminished immune responses to single S-antigen HBV vaccines [43]. A comparative study showed that among subjects aged ≥18 years, the seroprotection rate in the PreHevbrio group was 91.4%, compared to 76.5% in the Engerix-B group. In subjects aged ≥45 years, the seroprotection rate was 89.4% in the PreHevbrio group versus 73.1% in the Engerix-B group [45]. These findings indicate that PreHevbrio elicits superior immunogenicity compared to single S-antigen vaccines in adults. Similar results have been observed in HIV-positive adults without HBV infection. Following three doses of PreHevbrio vaccination, 84% of HIV-positive adults achieved seroprotection [46]. In patients with end-stage renal disease, HBV infection remains a significant epidemiological concern. Standard vaccination programs using single S-antigen vaccines achieve seroprotection in only 50–60% of this population. Among 29 end-stage renal disease patients who previously failed to respond to standard single S-antigen vaccination, 86% achieved seroprotective anti-HBs levels after receiving three doses of PreHevbrio [47]. Although these studies involve small sample sizes, the results still bring hope for those who have a poor response to conventional single S-antigen vaccines.

#### 3.2.4. Novel Adjuvant Hepatitis B Vaccine

The incorporation of various adjuvants has been shown to significantly enhance the immunogenicity of vaccines, thereby improving both the magnitude and duration of antibody responses. Traditionally, aluminum salts were the primary adjuvants utilized in human vaccines. However, in recent years, there has been a growing focus on novel adjuvants in vaccine research and development, particularly to address the suboptimal immune responses observed in specific populations, including individuals with chronic kidney disease, chronic liver disease, diabetes, and the elderly.

A novel adjuvant system AS04, composed of aluminum salts and the TLR4 agonist MPL (3-O-desacyl-4′-monophosphoryl lipid A), promotes antigen presentation to T cells and thereby increases immunogenicity by enhancing interactions between antigens and macrophages [48]. Fendrix is a hepatitis B vaccine formulated with the AS04 adjuvant system. It is indicated for individuals aged 15 years and older, and is primarily used in patients with kidney problems. The vaccination schedule consists of four doses administered within six months; the second, third, and fourth doses are given at intervals of one month, two months, and six months after the first dose, respectively [49]. A clinical trial conducted in patients undergoing hemodialysis as well as those prior to initiating hemodialysis demonstrated that, compared with the traditional recombinant hepatitis B vaccine, Fendrix achieved significantly higher seroprotection rates at 30 months (85% vs. 63%), 36 months (80% vs. 51%), and 42 months (78% vs. 52%) post-vaccination, with a significantly slower decline in seroprotection over time. This indicates that fewer patients may require booster doses [48]. Additionally, Fendrix has been shown to provide enhanced immune protection in patients with chronic liver disease [50].

Heplisav-B is a novel hepatitis B vaccine that incorporates synthetic cytosine phosphoguanine oligonucleotide (CpG1018), derived from bacterial DNA, as an adjuvant. It activates the Toll-like receptor 9 (TLR-9) signaling pathway to stimulate the immune system, thereby inducing the production of multiple cytokines. It is a two-dose vaccine administered one month apart and licensed in 2017 for use in adults aged 18 years and older [51]. Four randomized controlled trials involving 7056 recipients receiving two doses of Heplisav-B and 3214 recipients receiving three doses of Engerix-B demonstrated that the seroprotection rate (anti-HBs ≥ 10 mIU/mL) was significantly higher in the Heplisav-B group compared to the Engerix-B group (90–100% vs. 71–90%). Furthermore, Heplisav-B exhibited superior seroprotection rates in individuals known to have poor responses to traditional vaccines, including elderly individuals, patients with diabetes, and those with chronic kidney disease [52].

### 3.3. Therapeutic Hepatitis B Vaccines

**Figure 1:**
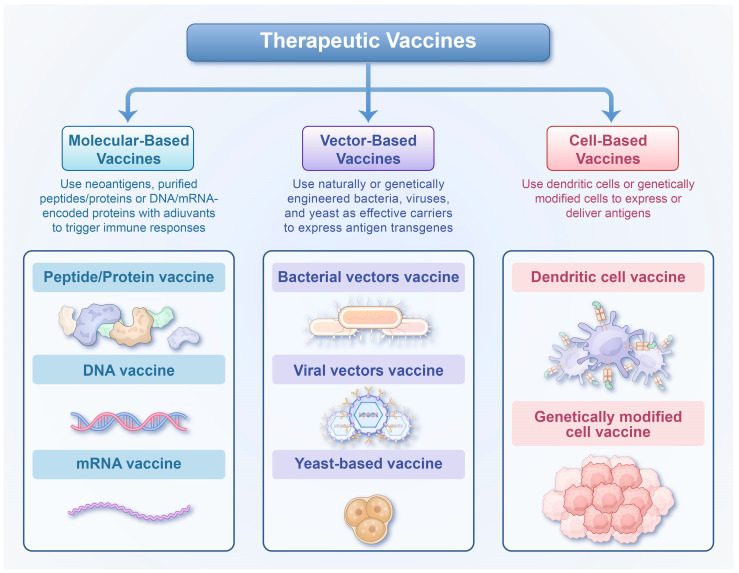
An overview of therapeutic vaccines. Therapeutic vaccines encompass several types currently utilized in preclinical and clinical development, including molecular-based vaccines (peptide/protein vaccine, DNA and mRNA vaccine), vector-based vaccines (bacterial vectors vaccine, viral vectors vaccine and yeast-based vaccines) and cell-based vaccines (dendritic cells vaccines and genetically modified cell vaccines).

In addition to develop the preventive hepatitis B vaccines aimed at enhancing immunogenicity, research on therapeutic hepatitis B vaccines for patients with chronic hepatitis B has also advanced significantly. The success of preventive vaccination relies on the rapid neutralization of invading pathogens through antibody-mediated mechanisms, whereas control and elimination of persistent viral infections require broad, multi-specific, and multi-functional effector T cell responses. The design principle of therapeutic vaccines involves the administration of non-infectious viral antigens to stimulate the host to generate de novo or enhance pre-existing pathogen-specific immune responses, ultimately aiming to control or eliminate the virus [53]. Therapeutic vaccines are primarily categorized into three types: molecular-based, vector-based, and cell-based vaccines [54] (Figure 1). Over the past decade, substantial progress has been made in the development of therapeutic vaccines against HBV; however, only a limited number have progressed to clinical trials, and to date, no strategy has successfully achieved functional cure.

BRII-179, also known as VBI-2601, is a therapeutic recombinant vaccine based on virus-like particles (VLPs) and derived from the preventive vaccine PreHevbrio. It contains three HBV surface envelope proteins, such as Pre-S1, Pre-S2, and S which are designed to enhance immune responses in patients with CHB. A clinical trial (ACTRN12619001210167) conducted in CHB patients receiving nucleos(t)ide analogue therapy demonstrated that administration of BRII-179 alone or in combination with low-dose interferon-α (IFN-α) led to increased antibody titers ranging from 2.1 to 998.8 mIU/mL, along with enhanced HBV-specific T cell responses. Although BRII-179 induced detectable humoral and cellular immune responses in a substantial proportion of participants, no significant correlation was observed between these immune responses and reductions in serum HBsAg levels. Throughout the study, no notable changes were observed in circulating HBV RNA or HBcrAg levels across any treatment group [55]. Despite the absence of viral load reduction, anti-Pre-S1 and anti-Pre-S2 antibody were detected by ELISA-based qualitative assays in patients receiving the combination of BRII-179 and IFN-α [55], indicating that IFN-αmay act as an immunomodulatory adjuvant. Furthermore, the study suggested that the limited antiviral efficacy of BRII-179 could be attributed to high baseline HBsAg levels and insufficient dosing. To address this, a follow-up study (NCT04749368) evaluating the combination of HBV-specific small interfering RNA (siRNA) with BRII-179 was initiated. Results indicated that siRNA-mediated reduction of HBsAg may facilitate the durability and potency of BRII-179-induced adaptive humoral immunity [56].

NASVAC is a therapeutic hepatitis B vaccine that contains HBsAg and hepatitis B core antigen (HBcAg). It was evaluated in a Phase III clinical trial (NCT01374308) involving patients with CHB who had serum HBV DNA levels ≥ 10^3^ copies/mL. After six months of treatment, the HBeAg seroconversion rate and the proportion of patients with undetectable HBV DNA were significantly higher in the NASVAC group compared to those treated with pegylated interferon (Peg-IFN): 35.7% vs. 18.7% and 57.7% vs. 35.0%, respectively [57]. Two years post-treatment, 50% of patients who received NASVAC still maintained undetectable HBV DNA levels [58], indicating a sustained antiviral effect. Furthermore, NASVAC is administered via the nasal route, offering a novel approach to vaccine delivery and potentially informing future developments in alternative administration strategies.

εPA-44 is a liposome-based nanoparticle lipopeptide vaccine. Its immunogen consists of a linear single-chain polypeptide comprising 44 amino acid residues, composed of a dominant protective HLA-A2 restricted cytotoxic T lymphocyte (CTL) epitope derived from HBcAg, a Th1 helper T cell epitope from tetanus toxoid, a B-cell epitope from the Pre-S(2) region (amino acids 133–143) of HBsAg, and a palmitic acid molecule serving as a adjuvant. A randomized clinical trial (NCT00869778) conducted in leukocyte antigen A2 (HLA-A2)-positive and HBeAg-positive patients demonstrated that the εPA-44 group achieved a significantly higher HBeAg seroconversion rate compared to the placebo group (38.8% vs. 20.2%). Additionally, the combined endpoint of HBeAg seroconversion, alanine aminotransferase (ALT) normalization, and HBV DNA levels below 2000 IU/mL for both receiving 900 µg εPA-44 (18.1%) and receiving 600 µg εPA-44 (14.3%) resulted in a significantly higher rate versus the placebo group (5.0%) [59].

Research on therapeutic nucleic acid vaccines for HBV is ongoing. However, to date, no mRNA-based vaccines have entered clinical trials, despite the advancement of numerous DNA vaccine candidates into clinical stages, with outcomes thus far being suboptimal. The pCMV-S2.S DNA vaccine encodes the HBV surface envelope proteins pre-S2 and S. Clinical trial results (NCT00536627) indicate that although this HBV DNA vaccine is well tolerated, it fails to restore functional anti-HBV immune responses following discontinuation of nucleos(t)ide analogues therapy [60]. Another dual-plasmid DNA vaccine consists of the pS2.S plasmid (pcDNA3.1+/S2.S), which encodes the HBV surface envelope protein S, and the pFP adjuvant plasmid (pcDNA3.1-/IL2 + IFN-γ), which expresses a fusion protein of interleukin-2 (IL-2) and interferon-γ (IFN-γ). This formulation enhances local antigen presentation by antigen-presenting cells, thereby improving immunogenicity. Results from a clinical trial (NCT01487876) demonstrated that patients receiving in vivo electroporation (EP)-mediated dual-plasmid HBV DNA vaccine in combination with lamivudine (LAM) achieved a significantly higher HBeAg seroconversion rate compared to controls (54.5% vs. 15.8%); however, no significant reduction in HBV DNA levels was observed [61].

TG1050 is an adenovirus 5-based vaccine that expresses polymerase, HBcAg, and HBsAg. It is capable of inducing HBV-specific T cell responses. Results from the NCT02428400 clinical trial indicate that in patients with CHB receiving nucleos(t)ide analogue therapy, the addition of TG1050 does not lead to significant reductions in HBsAg or HBcAg levels in most patients [62].

GS-4774 is a heat-inactivated, yeast-based T cell therapeutic vaccine that expresses HBV S, X, and core proteins. It is designed to elicit robust HBV-specific lT cell responses. Results from a Phase II clinical trial (NCT01943799) demonstrated that GS-4774 was safe and well tolerated in patients with chronic HBV infection receiving oral antiviral therapy; however, it did not reduce serum HBsAg levels [63]. In a separate study (NCT02174276), when administered in combination with tenofovir disoproxil fumarate (TDF), GS-4774 was shown to improve the production of interferon-γ (IFN-γ), tumor necrosis factor (TNF), and interleukin-2 (IL-2) by HBV-specific CD8+ T cells, suggesting its potential to enhance immune responses when combined with antiviral agents [64].

In the initial phases of research, numerous therapeutic vaccines for HBV were subjected to clinical trials; however, the results were predominantly disappointing. The findings from these trials indicate that researchers have moved beyond the evaluation of HBV therapeutic vaccines as standalone treatments. Instead, there has been a growing emphasis on investigating innovative strategies aimed at enhancing therapeutic efficacy. Several of these novel approaches have demonstrated potential in augmenting HBV-specific immune responses and decreasing viral load.

### 3.4. Challenges of Hepatitis B Vaccine Administration

With the increasing global emphasis on HBV vaccination, as of the end of 2024, 190 WHO member states have implemented universal routine HBV vaccination for infants, achieving a global three-dose vaccine coverage rate of 84%. Furthermore, 117 member states have administered the first dose within 24 h after birth, resulting in a global coverage rate of 45%. However, substantial regional disparities persist: coverage in the Western Pacific Region reaches 79%, whereas in the African Region, it is estimated at only 17% [39]. These discrepancies are associated with regional economic conditions, as well as variations in public awareness of hepatitis B risks and understanding of vaccination guidelines, including perceptions of vaccine safety and efficacy. In countries that have implemented the universal routine HBV vaccination program, data indicate a decline in coverage since 2019, likely attributable to the COVID-19 pandemic, associated public health interventions, and increased parental hesitancy toward vaccination [65]. To address gaps in protection, it is essential to revise vaccination recommendations and implement catch-up strategies for older children and adolescents who missed early vaccination during infancy. Additionally, a survey of healthcare professionals reveals that while most healthcare professionals are aware of and adhere to national vaccination recommendations, many do not actively recommend hepatitis B vaccination for high-risk populations, perceiving their risk to be low. The survey also highlights out-of-pocket costs as a significant barrier preventing high-risk individuals from accessing hepatitis B vaccination [66].

Vaccine responses are classified according to anti-HBs antibody titers into three categories: non-responders (<10 IU/L), responders (10–100 IU/L), and high responders (≥100 IU/L). Although most healthy individuals who complete standard vaccination achieve at least 20 years of protection from initial immunization, approximately 5–10% of vaccine recipients fail to develop an adequate immune response. Age, smoking, and high body mass index (BMI) are well-established risk factors associated with non-response [67]. Additional variables such as gender and the presence of concomitant diseases, such as diabetes, chronic kidney disease, and HCV infection, also contribute to diminished immune responses to the hepatitis B vaccine [68,69]. In individuals infected with human immunodeficiency virus (HIV), seroconversion rates after hepatitis B vaccination vary significantly depending on immune status, ranging from 18% to 72% [70]. Traditional recombinant surface (S) antigen vaccines exhibit substantially reduced immunogenicity in adults, older populations, and individuals with chronic diseases. Following completion of the standard 3-dose vaccination, fewer than 75% of vaccinated individuals achieve protective levels of HBV antibodies (anti-HBs ≥ 10 mIU/mL) [43].To address variability in vaccine response due to host-related factors, vaccination strategies can be optimized to enhance immunogenicity through approaches such as using vaccines with higher immunogenicity, increasing the number of doses, or administering higher antigen doses. The novel adjuvanted vaccine HEPLISAV-B, designed to improve immunogenicity, has demonstrated improved response rates in populations including HIV-positive individuals, older adults, and patients with diabetes [71]. For immunocompromised individuals, such as those with chronic kidney disease or on dialysis, type 2 diabetes, organ transplant recipients, chronic liver disease, inflammatory bowel disease, HIV infection, or various cancers, increasing the dose frequency or antigen dosage significantly improves seroprotection rates [72].

The implementation of universal hepatitis B vaccination programs has played a crucial role in the management and reduction of HBV infections. Nonetheless, considerable obstacles persist in realizing the WHO’s objective of eradicating hepatitis B as a public health threat by the year 2030. To meet this elimination target, future initiatives must prioritize the expansion of vaccine coverage, address regional disparities in healthcare access, and promote the research and development of therapeutic vaccines.

## 4. Hepatitis C Vaccine

### 4.1. Hepatitis C

WHO estimates that approximately 50 million people worldwide are chronically infected with HCV, and about 1 million new infections occur annually. In 2022, an estimated 242,000 deaths were attributed to hepatitis C [73]. Although direct-acting antivirals (DAAs) are currently available, with cure rates exceeding 95%, short treatment durations, and minimal side effects, their high cost limits widespread accessibility, particularly in regions with limited medical resources. Furthermore, following successful DAA treatment, patients do not develop protective immunity, leaving them susceptible to reinfection [74]. The early stage of HCV infection is often asymptomatic, and many infected individuals remain unaware of their condition, facilitating silent transmission of the virus. It is estimated that 55% to 85% of infected individuals will progress to chronic infection [73].

HCV is a blood-borne pathogen. Common routes of transmission include reuse of contaminated medical equipment or inadequate sterilization, transfusion of unscreened blood or blood products, and injection drug use involving shared syringes. HCV can also be transmitted through mother-to-child transmission and sexual contact involving exposure to blood, although these modes are less frequent [75]. Evidence indicates that MSM and PLWH are at high risk for sexually acquired HCV infection. A prospective study of acute HCV cases diagnosed at a hospital on Gran Canaria Island between 2016 and 2020 found that 83.3% of HCV-infected individuals reported male-to-male sexual activity, 68.2% were HIV positive, and 31.8% had a history of drug use [76].

Chronic hepatitis C represents a significant etiological factor in the development of liver cirrhosis and HCC, thereby imposing considerable health and economic burdens on patients, their families, and society at large. Consequently, the development of an effective vaccine is regarded as the most cost-effective strategy for fundamentally disrupting viral transmission and ultimately achieving the elimination of hepatitis C. However, since the first isolation of HCV in 1989, researchers have been exploring the possibility of developing a vaccine, but so far, no widely effective vaccine has been successfully developed [73].

### 4.2. Development and Challenges of the Hepatitis C Vaccine

Following HCV infection, a complex innate and adaptive immune response will occur in the body. 15% to 45% of infected individuals are able to spontaneously clear the virus within a few months and do not progress to chronic or lifelong infection. Among these individuals, 80% can also spontaneously clear reinfection [77]. This unique immunological feature, rarely observed in other chronic viral infections, offers promising evidence for the feasibility of developing an effective HCV vaccine.

HCV primarily infects humans and chimpanzees. However, due to ethical constraints, chimpanzees are no longer routinely used in experimental research. Because of the narrow host specificity of HCV, developing practical small animal models, such as laboratory mice and rats, for HCV infection remains highly challenging. The lack of robust immunocompetent animal models for HCV impedes vaccine development and studies of immune responses [78]. A major breakthrough occurred in 2005 with the identification of the full-length HCV isolate JFH1, which was capable of replicating the entire viral life cycle in the human hepatoma cell line Huh7, known as cell culture-derived hepatitis C virus (HCVcc), and successfully constructed a humanized mouse model. By incorporating human liver cells, this model is capable of partially replicating HCV infection process as it occurs in humans. Consequently, it has made progress in addressing the persistent challenge of the absence of appropriate animal models for HCV research [79].

**Figure 2:**
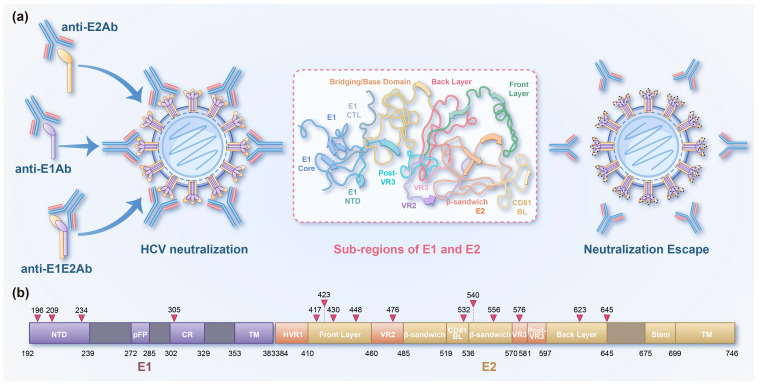
HCV evades host-derived neutralization through glycan shielding. (**a**) Mechanism diagram of HCV evading neutralizing antibody (nAb) responses. Broadly cross-reactivity neutralizing antibodies may limit viral immune escape by targeting highly conserved epitopes. The primary targets include envelope glycoproteins E1 and E2, which are exposed on the viral surface and form a functional E1E2 heterodimer. The extracellular domains of the envelope glycoproteins E1 and E2 are highly glycosylated, resulting in the physical masking of critical neutralizing epitopes. This glycan-mediated shielding limits the accessibility of neutralizing antibodies to E1, E2 and E1E2 heterodimer, thereby enabling the virus to evade humoral immune responses. (**b**) Domain organization of HCV envelope glycoproteins. On E1, key structural elements include the N-terminal domain (NTD), putative fusion peptide (pFP), the conserved region (CR) and the transmembrane (TM) regions. The hypervariable region 1 (HVR1), variable regions 2 and 3 (HVR2 and HVR3), the front layer, the back layer, the β-sandwich domains, stem and the transmembrane regions are labelled on E2. In most HCV genotypes, E1 contains four conserved N-linked glycosylation sites located at amino acid positions 196, 209, 234, and 305. E2 typically harbors up to eleven N-linked glycosylation sites, positioned at residues 417, 423, 430, 448, 476, 532, 540, 556, 576, 623, and 645. Glycosylation sites are marked with a red arrow.

One of the major challenges in developing an HCV vaccine is the viral high genetic diversity (Figure 2). HCV comprises eight major genotypes and 86 subtypes. Sequence variation among subtypes within the same genotype can range from 15% to 25%, while variation between different genotypes may reach up to 30% [80]. Researchers worldwide are actively pursuing the development of a single, universal hepatitis C vaccine. The HCV genome is approximately 9.6 kilobases (kb) in length and encodes a polyprotein that is subsequently processed into structural proteins and non-structural proteins. Structural proteins contain the core protein (Core), envelope glycoprotein 1 (E1), and envelope glycoprotein 2 (E2), while non-structural proteins contain p7, NS2, NS3, NS4A, NS4B, NS5A, and NS5B [81]. Both the core protein and non-structural proteins are present in the virus particles, while only the envelope glycoproteins E1 and E2 are localized on the viral surface, where they are easily recognized by antibodies. E1 and E2 dimerize to form the E1E2 complex, which is present on the surface of the virus and serves as the primary target for neutralizing antibodies [82]. The Chiron E1E2 HCV vaccine is an envelope protein-based candidate vaccine, containing a recombinant E1E2 glycoprotein derived from the HCV 1a genotype and the adjuvant MF59C.1, a water-in-oil emulsion. In its Phase I clinical trial (NCT00500747), the vaccine demonstrated favorable safety and tolerability, and induced E1E2-specific CD4+ T cell proliferation along with the production of E1E2-specific cytokines [83]. Although this vaccine has demonstrated safety and immunogenicity, further research is required to evaluate its efficacy.

In the development of HCV vaccines, in addition to humoral immune responses mediated by broadly neutralizing antibodies (bNAbs), T cell-mediated immunity should also be considered. HCV-specific CD4+ and CD8+ T cells play a crucial role in virus clearance by targeting multiple viral antigens, with non-structural (NS) proteins representing a primary target [84]. A T cell vaccine based on viral vector delivery of encoded non-structural proteins has been evaluated in a clinical trial (NCT01436357). A heterologous prime-boost vaccination strategy was administered to injection drug users. The initial dose consisted of a recombinant chimpanzee adenovirus 3 (ChAd3) vector encoding ChAd3-NSmut1, which delivers the NS3-NS5B region of the HCV genotype 1b genome. Eight weeks later, a booster vaccination was administered using the modified vaccinia Ankara (MVA) vector expressing the same NS3-NS5B sequence as MVA-NSmut. The results showed that this vaccination strategy did not prevent HCV infection. However, when assessing immunogenicity prior to HCV exposure, 78% of participants in the vaccine group exhibited HCV-specific T cell responses, significantly higher than the 3% observed in the placebo group. Furthermore, monitoring of geometric mean HCV RNA levels revealed that viremia increased after infection in the placebo group, peaking one month post-infection, whereas in the vaccine group, the geometric mean peak HCV RNA level occurred at the time of infection, with no subsequent rise in post-infection viremia [85].

The immune evasion mechanism of HCV poses great challenges to vaccine development. The two primary mechanisms by which HCV evades the host immune system are the high variability of the amino acid sequence and epitope shielding through glycosylation of the envelope proteins [86]. The HCV RNA polymerase, NS5B, lacks proofreading activity, making the virus highly error-prone during genome replication. Coupled with its rapid replication, a large number of viral sequence variants can be generated daily within the infected host, thereby enabling effective immune escape [87]. The extracellular domain of the HCV envelope glycoprotein E1 and E2 are highly glycosylated. In most genotypes, E1 contains four conserved glycosylation sites, while E2 harbors up to eleven. The envelope glycoprotein is modified by N-linked glycans, masking the conserved neutralizing epitopes on the surface of HCV particle and reducing the immunogenicity of the envelope proteins. Moreover, glycans have been demonstrated to play a key role in immune evasion by shielding antigenic sites targeted by neutralizing antibodies [88] (Figure 2). Additionally, HCV particles can associate with lipoproteins and apolipoproteins in the host to form lipo-viral particles (LVPs). When HCV exists in the form of LVPs, apolipoproteins on the particle surface can mask the viral epitopes, enabling the virus to evade neutralizing anti-HCV antibodies and enhancing its resistance to the humoral immune response [89]. A comprehensive understanding of the mechanisms by which HCV evades the host immune response, including glycans and LVPs, is an essential parameter to take into account in the design of an HCV vaccine.

## 5. Hepatitis D Vaccine

Hepatitis D is a severe liver disease caused by HDV. It typically occurs as a co-infection or superinfection alongside HBV, increasing the risk of developing liver cirrhosis and hepatocellular carcinoma. HDV is a defective virus that lacks the ability to encode its own envelope proteins and therefore depends on the capsid proteins of HBV for viral assembly, replication, and transmission [90]. After acute co-infection with both HDV and HBV, approximately 95% of immunocompetent individuals are able to clear both viruses spontaneously. However, in patients already chronically infected with HBV who acquire HDV as a superinfection, more than 90% progress to chronic HDV-HBV infection. Chronic hepatitis D accelerates the progression of liver disease compared to HBV monoinfection, with studies indicating that approximately 30% to 70% of patients having developed liver cirrhosis at the time of diagnosis, and over 50% die from liver related complications within ten years post-diagnosis [91]. In high-income countries, the extensive implementation of hepatitis B vaccination programs has resulted in effective control ofHDV, leading to a shift in the clinical epidemiology toward older populations with advanced fibrosis or cirrhosis. Conversely, in low- and middle-income countries, where coverage of hepatitis B vaccination remains inadequate, HDV continues to represent a substantial public health challenge [91]. Consequently, the vaccination against hepatitis B is recognized as the most effective strategy for preventing HDV infection.

## 6. Hepatitis E Vaccine

### 6.1. Hepatitis E

HEV represents the predominant etiological agent of acute viral hepatitis and is categorized into eight distinct genotypes. Among these, genotypes HEV1 to HEV4 are chiefly implicated in human infections. Genotypes HEV1 and HEV2 are predominantly associated with epidemic occurrences in developing nations, primarily transmitted via contaminated water sources, and frequently result in extensive outbreaks. Conversely, genotypes HEV3 and HEV4 are characterized as zoonotic, utilizing a diverse array of animal reservoirs such as pigs, boars, rabbits, and deer. Infections with these genotypes primarily occur in high-income countries and are typically linked to the consumption of raw or undercooked pork or game meat [92]. The majority of HEV infections are self-limiting and recover spontaneously within a few weeks without specific intervention. However, prolonged infection may occur in immunocompromised individuals who fail to clear the virus, potentially progressing to chronic hepatitis [93]. Although hepatitis E is generally mild in immunocompetent populations, it can lead to severe clinical outcomes, including fulminant hepatic failure. Pregnant women, particularly during the second or third trimesters, face a markedly elevated risk of acute liver failure, miscarriage, and death, with case mortality reaching from 20% to 25% in late pregnancy. According to global estimates, there were approximately 19.47 million cases of acute hepatitis E in 2021, resulting in around 3450 deaths [94]. To date, no antiviral therapy has been specifically approved for the treatment of HEV infection; consequently, preventive measures, particularly vaccination, represent the primary strategy for controlling HEV transmission.

### 6.2. Types of Hepatitis E Vaccine

The recombinant hepatitis E vaccine (rHEV) was initially developed by the National Institutes of Health (NIH) in the United States in collaboration with GlaxoSmithKline (GSK). This vaccine consists of a purified polypeptide expressed in Spodoptera frugiperda cells infected with a recombinant baculovirus, containing the HEV1 genomic sequence that encodes the capsid antigen. A Phase II clinical trial (NCT00287469) was completed, and the results indicated a vaccine efficacy of 95.5% following administration of three doses [95]. However, due to various factors, including commercial viability and market considerations, GSK discontinued further research and development of this vaccine.

Additionally, two other HEV vaccines developed in China, HEV p179 and HEV 239 (Hecolin), have entered clinical trials. HEV p179 is produced by inserting the p179 gene fragment encoding amino acids 439–617 of HEV4 ORF2 into the plasmid pET28a. Recombinant proteins are then expressed in *Escherichia coli* *E. coli* BL21 and assembled into virus-like particles (VLPs). Although this candidate vaccine completed a Phase I clinical trial and demonstrated favorable safety and tolerability, further development was discontinued [96]. Currently, HEV 239 (Hecolin) is the only licensed HEV vaccine available globally. This vaccine utilizes recombinant genetic engineering technology to efficiently express the ORF2 protein (amino acids 368–606) of HEV1 in *Escherichia coli*, followed by assembly into VLPs. These VLPs structurally mimic native HEV virions, enabling them to elicit robust protective immune responses [97]. The HEV 239 (Hecolin) vaccine was approved for use in China in 2011. The target population for vaccination mainly includes individuals aged 16 years and older, with particular recommendations for high-risk groups. A three-dose vaccination schedule (0-1-6) is implemented, with the second dose administered one month after the first and the third dose administered six months after the first. Results from the Phase III clinical trial (NCT01014845) demonstrate that the HEV 239 (Hecolin) vaccine exhibits excellent safety, efficacy, and immunogenicity in healthy adults aged 16–65 years [98]. Notably, although the vaccine is based on a genotype 1 strain, the majority of circulating HEV strains in the trial population were genotype 4, indicating cross-genotype protective immunity. A ten-year follow-up of participants in the Phase III clinical trial revealed that the antibodies induced by the HEV 239 (Hecolin) vaccine persisted for at least 8.5 years, with a geometric mean concentration of 0.20 U/mL remaining after 10 years [99]. Due to insufficient data on safety, immunogenicity, and efficacy in certain populations, WHO does not currently recommend routine vaccination with HEV 239 (Hecolin) in children under 16 years of age, pregnant women, patients with chronic liver disease, individuals on the organ transplant waiting list, or international travelers [100].

### 6.3. Challenges of Hepatitis E Vaccine Administration

Currently, only the HEV 293 vaccine has been approved for clinical use; however, its application is subject to several limitations. Beyond China and Pakistan, HEV 293 has not been approved in other countries, highlighting the need to accelerate international clinical trials to evaluate its safety and immunogenicity. Furthermore, HEV 293 is licensed exclusively for individuals aged 16 years and older. However, a study conducted in South Sudan indicated that over 70% of PCR-confirmed hepatitis E cases occurred in individuals under 16 years old, precisely the population excluded from current vaccination eligibility. This finding underscores the critical need to conduct clinical trials assessing the safety and efficacy of HEV 239 (Hecolin) in children and adolescents, aiming to expand protection for these vulnerable groups [101].

Given the severe consequences of HEV infection in pregnant women, evaluating the safety and efficacy of the HEV 239 (Hecolin) vaccine in this population has consistently been a focal point. In Bangladesh, after non-pregnant women aged 16–39 were vaccinated with HEV 239 (Hecolin) and monitored weekly through pregnancy testing, it was found that the risk of miscarriage increased among subjects who were vaccinated within 90 days before pregnancy or inadvertently vaccinated during early pregnancy, with rates of 8.9% and 10.5% respectively [102]. Conversely, a phase 3 clinical trial in China reported different findings, demonstrating that participants in the vaccine group exhibited similar maternal and neonatal safety profiles comparable to those in the control group, regardless of the maternal age [103]. In light of these conflicting results, further assessment of the risk of spontaneous abortion after HEV vaccination before and after conception and in the early stages of pregnancy is warranted.

Moreover, studies have evaluated the safety and immunogenicity of HEV 293 in patients with CHB. The results showed that both the seroconversion rate and the geometric mean concentration (GMC) of anti-HEV IgG in the CHB group were similar to those observed in the healthy adult group, with no serious vaccine-related adverse events were reported [104].

Overall, there is limited available data on the vaccination of special populations with hepatitis E vaccine. The safety and immunogenicity of this vaccine when administered in combination with other vaccines have not been fully evaluated, necessitating further clinical trials. Additionally, expanding vaccine coverage is crucial for reducing the infection rate. WHO prequalification must be pursued to broaden the global applicability and accessibility of HEV 239.

## 7. Combination Hepatitis Vaccines

The development of combination vaccines aims to prevent multiple infectious diseases with fewer vaccination doses while maintaining high standards of safety and efficacy. Currently, several combination hepatitis vaccines are available globally: the combined hepatitis A and B vaccine (Ambirix, Twinrix), the combined hepatitis A and typhoid vaccine (Viatim/Vivaxim and Hepatyrix) [5], and the hexavalent vaccine Hexaxim (DTaP-IPV-HB-Hib), which provides protection against diphtheria, tetanus, pertussis, polio, hepatitis B, and invasive diseases caused by Haemophilus influenzae type b [105].

The use of combination vaccines has greatly enhanced vaccination efficiency and coverage, while reducing medical costs. The combined hepatitis A and B vaccine (HepAB) typically follows a three-dose immunization schedule: the first dose may be administered at any time, followed by the second dose one month later and the third dose six months after the initial dose. Studies have shown that 12–15-year-old children receiving two doses of HepAB (Twinrix) and adults receiving three doses can achieve high levels of antibody protection that may last for several decades [106]. The combined hepatitis A and typhoid vaccine is usually administered as a single dose to travelers aged 16 and older, especially before traveling to areas where these diseases are prevalent. A large amount of data has proved that these two combination hepatitis A and typhoid vaccines can provide more convenience and rapid seroconversion for travelers [107,108]. The hexavalent vaccine Hexaxim is used in the European Union for primary and booster vaccinations in infants over 6 weeks of age, with at least two doses spaced 8 weeks apart or at least three doses spaced 4 weeks apart, and a booster dose can be given at least 6 months after completion of the primary series [109]. Hexaxim was first approved in 2012 and has been widely adopted in the 10 years following its licensing. Over 180 million doses of Hexaxim have been distributed globally, demonstrating favorable safety and high immunogenicity [105].

## 8. Conclusions

Over recent decades, vaccines have been instrumental in advancing the objective of controlling viral hepatitis. The enhancement of hygiene conditions, coupled with the implementation of hepatitis A vaccination initiatives, has led to a global decline in the incidence of hepatitis A, particularly in nations that have adopted the Universal Childhood Vaccination Program (UCVP). Nonetheless, outbreaks persist among high-risk groups not encompassed by the UCVP, underscoring the necessity to broaden vaccine coverage. Although the improvement of sanitation is vital for diminishing or eradicating hepatitis A transmission, vaccination continues to serve as an indispensable complementary strategy. The implementation of the universal hepatitis B vaccination program has significantly contributed to the control of HBV infections. Future challenges in the development of hepatitis B vaccines include the enhancement of immunogenicity for individuals who demonstrate suboptimal responses to existing vaccines, as well as the expedited development of therapeutic vaccines for patients with chronic hepatitis B, with the overarching objective of achieving HBV elimination. In contrast, while an effective vaccine for the prevention of HCV infection is currently unavailable, ongoing scientific endeavors have resulted in numerous clinical trials assessing candidate hepatitis C vaccines. It is anticipated that a safe and highly immunogenic HCV vaccine will be developed in the near future. At present, there is no dedicated vaccine available for the prevention of HDV infection. Nevertheless, the extensive administration of the hepatitis B vaccine has significantly reduced the transmission of HDV, due to the dependency of HDV replication on co-infection with HBV. Although a safe and efficacious vaccine for HEV is available, its licensure is currently restricted to China and Pakistan. Considering the global prevalence of HEV and its capacity to trigger large-scale outbreaks, especially in low-income regions, there is an urgent necessity to develop and distribute an HEV vaccine that is suitable for global application.

## Figures and Tables

**Table 1:** Monovalent hepatitis A vaccines are available globally.

Type	Trade Name	Year Licensed	HAV Strain	Adjuvant	Manufacturers
HepA-I	Havrix	1992	HM-175	Aluminium hydroxide	GlaxoSmithKline Biologicals, London, UK
	Vaqta	1993	CR-326	Aluminium hydroxide	Merck, Sharp & Dohme Corporation, Kennywood, NJ, USA
	Avaxim	1996	GBM	Aluminium hydroxide	Sanofi Pasteur, Paris, France
	Epaxal	1997	RG-SB	Virosome	Crucell/Berna Biotech/Janssen Cilag Ltd., Bern, Switzerland
	Healive	2002	TZ84	Aluminium hydroxide	Sinovac Biotch Co., Ltd., Beijing, China
	Weisairuian	2006	Lv-8	Aluminium hydroxide	Institute of Medical Biology of the Chinese Academy of Medical Sciences, Kunming, China
	Veraxim	2009	YN5	Aluminium hydroxide	Shanghai Wilson Bioengineering Inc., Shanghai, China
	Aimmugen	1994	KRM003	Aluminium-free	KM Biologics Co., Ltd., Kumamoto, Japan
HepA-L	Weisairuiji (Freeze-dried)	2003	H2	None	Institute of Medical Biology of the Chinese Academy of Medical Sciences, Kunming, China
	Freeze-dried live attenuated hepatitis A vaccine	2003	H2	None	Zheijiang Pukang Biotechnology Company Limited, Zhejiang, China; Academy of Medical Sciences, Hangzhou, China
	Havac Weisairuiji (Freeze-dried)	2000	LA-1	None	Changchun Institute of Biological Products, Changchun, China

Note: Inactivated hepatitis A vaccine, HepA-I; live attenuated hepatitis A vaccine, HepA-L.
